# A Single-Unit Design Structure and Gender Differences in the Swimming World Championships

**DOI:** 10.2478/hukin-2014-0075

**Published:** 2014-10-10

**Authors:** Svetlana Pushkar, Vladimir B. Issurin, Oleg Verbitsky

**Affiliations:** 1 Department of Civil Engineering, Faculty of Engineering, P.O.B. 3, Ariel University, Ariel, Israel.; 2 Elite Sport Department, Wingate Institute of Physical Education & Sport, Netanya, Israel.; 3 Faculty of Biomedical Engineering, Technion, Israel Institute of Technology, Haifa, Israel.

**Keywords:** Clusters, designing studies, statistical analysis, nondemonic intrusion, sample size, sacrificial pseudoreplication

## Abstract

Four 50 meter male/female finals - the freestyle, butterfly, breaststroke, and backstroke - swum during individual events at the Swimming World Championships (SWCs) can be defined in four clusters. The aim of the present study was to use a single-unit design structure, in which the swimmer was defined at only one scale, to evaluate gender differences in start reaction times among elite swimmers in 50 m events. The top six male and female swimmers in the finals of four swimming stroke final events in six SWCs were analyzed. An unpaired t-test was used. The p-values were evaluated using Neo-Fisherian significance assessments (Hurlbert and Lombardi, 2012). For the freestyle, gender differences in the start reaction times were positively identified for five of the six SWCs. For the backstroke, gender differences in the start reaction times could be dismissed for five of the six SWCs. For both the butterfly and breaststroke, gender differences in the start reaction times yielded inconsistent statistical differences. Pooling all swimmers together (df = 286) showed that an overall gender difference in the start reaction times could be positively identified: p = 0.00004. The contrast between the gender differences in start reaction times between the freestyle and backstroke may be associated with different types of gender adaptations to swimming performances. When the natural groupings of swimming stroke final events were ignored, sacrificial pseudoreplication occurred, which may lead to erroneous statistical differences.

## Introduction

Four 50 meter finals - the freestyle, butterfly, breaststroke, and backstroke - swum during individual events at the Swimming World Championships (SWCs) can be defined in four clusters or “natural grouping[s] of objects that may have similar attributes” (Picquelle and Mier, 2011). Similar situations have been comprehensively described in ecological field studies (Hurlbert, 1984; 2013) and in neuroscientific studies (Lazic, 2010). It is worth noting that clustered data arises commonly in sports research (Hayen, 2006). The protocols of the SWCs contain information about start reaction time (SRT) in seconds for elite male and female swimmers in 50 m final events (the Omega official timekeeper 2013 (http://www.omegatiming)). Consequently, based on the protocols of the SWCs, gender differences (GDs) in SRTs in naturally created groups can be obtained and investigated. Studying SRTs in elite athletes has a long history. Mero and Komi (1990) found that there was great inter-individual variation in patterns of muscle activation, thereby emphasizing the complexity of sprint starts. We hypothesize that GDs in SRTs may shed light on the differences played by gender adaptations on maximal performance among elite swimmers due to different swimming strokes. To test this hypothesis, each swimming stroke final event should be treated independently. Each event can be defined as a single-unit design structure in which the primary sampling unit or the experimental unit is defined at only one scale (Hurlbert, 2013).

Recently, GDs in SRTs have been analyzed in three observational studies (Brown et al., 2008; Pilianidis et al., 2012; Tønnessen et al. 2013) of which data sets included different event settings such as three Olympic Games (2000, 2004 and 2008), four Athletic World Championships (AWCs) (2003, 2005, 2007 and 2009) and four Athletic World Youth Championships (AWYCs) (2003, 2005, 2007 and 2009). The data sets also included different subtypes of races such as the 100 and 200 m sprints and the 100/110 m hurdles within one event setting. However, in those analytical studies, the effects of different event settings and/or different subtypes of races on gender differences in start reaction times were ignored. An analysis of these three studies is presented below. GDs in SRTs among athletic sprinters in the 2000, 2004, and 2008 Olympic Games were previously analyzed. SRTs were collected from athletes who took part in the final heats of the 100 m and 200 m races and also in the 110/100 m hurdles (Pilianidis et al., 2012). The results of the Pilianidis et al. (2012) study indicated that female sprinters had slower start reaction times in the 100 m sprint than the male athletes for the same distance in the 2000, 2004, and 2008 Olympic Games. For example, in the 2000 Olympic Games, the start reaction times for male sprinters and for female sprinters were reported as 180 ± 30 ms and 207 ± 32 ms, respectively, with an average difference of 27 ms or 15% (Pilianidis et al., 2012). In the 2004 Olympic Games, the start reaction times for male sprinters and for female sprinters were found to be 166 ± 12 ms and 185 ± 20 ms, respectively. The average difference was 19 ms or 11%. In the 2008 Olympic Games, the start reaction times for male sprinters and for female sprinters were 146 ± 14 ms and 183± 33 ms, respectively. The average difference was 43 ms or 25%. The authors of that study aggregated into one group with the same scale the three final events (viz., the 100 and 200 m sprints and the 100/110 m hurdles) (Pilianidis et al., 2012). Therefore, those authors ignored the individual properties of the elite athletes that were naturally divided across the three different races. GDs in SRTs were also studied for the 2004 Olympic Games (Brown et al., 2008). SRTs were compiled for both the male and female 100 m sprint and 100/110 m hurdles. SRTs were collected on 375 athletes (male and female). The results showed that, overall, male sprinters had significantly lower SRTs (163 ± 22 ms) than the female sprinters (188 ± 28 ms), with an average difference of 25 ms or 15%. In the Brown et al. (2008) study, athletes competing in different races (viz., the 100 m and the 100/110 m hurdles races) were pooled into one scale group. Therefore, those authors (Brown et al., 2008) also ignored the individual properties of the elite athletes that were naturally divided between the two different types of races. The relationship between GDs in SRTs was investigated among elite sprinters during four AWCs (2003 – 2009) and four AWYCs (2003–2009) (Tønnessen et al., 2013). Researchers found that the average SRT (166 ± 30 ms) of males was significantly shorter (p < 0.01) than that of females (176 ± 34 ms), with a difference of 11 ms or 6%. Tønnessen et al. (2013) used large sample sizes (males, *n* = 674; females, *n* = 540) to analyze reaction times at the start of races. Those authors suggested that the athletes who had participated in the four AWCs and the four AWYCs competed under the same conditions. However, the World Championships were performed in different geographic regions (Tønnessen et al., 2013).

Therefore, in the three aforementioned studies, there were three common statistical errors. The first statistical error was ignoring nondemonic intrusion, which is defined as “the impingement of chance events on an experiment in progress” (Hurlbert, 1984). The second statistical error was “ignoring the sampling structure in the analysis” (Picquelle and Mier, 2011). The third statistical error was a fallacy of large sample size (Hurlbert and Lombardi, 2009): as Hurlbert and Lombardi (2009) allegorically noted: “it lurks quietly in the darkness, waiting for researchers to pass by who are too focused on obtaining adequate sample sizes. If sample sizes are too large, one may be ‘in danger’ of getting very low p-values and establishing the sign and magnitude of even small effects with too much confidence.”

In this study, we advocate a way to minimize the problems associated with nondemonic intrusions, natural groupings of objects, and large sample sizes. For example, the different geographic locations and different constructions of the starting blocks at the SWCs between 2003 and 2013 can be conceptualized as non-demonic intrusions (Table 1). Different swimming strokes (i.e., a total of four stokes) and the top six places in final events at SWCs are related to natural groupings of swimmers. Evaluating each final event for a swimming stroke as an independent group is a remedy for the problem of large sample size. Therefore, we suggest that two swimmers in a single final event at the same SWC share more similar environmental conditions compared to the same two swimmers at two different SWCs. Similar suggestions have been made for biological studies (Kozlov and Hurlbert, 2006; Verbitsky, 2013) and research in sports medicine and sports science (Hayen, 2006). It was recently noted that when a biological variation in response to some intervention was the variable of interest in the analysis of samples, considering the natural grouping of objects was essential (Landis et al., 2012).

Therefore, the current study has three main goals: (i) to determine gender differences in start reaction times for four different 50 m stroke final events at six Swimming World Championships, (ii) to assess the effects of ignoring natural groupings at Swimming World Championships, and (iii) to evaluate the effects of the large sample size on statistical inferences when gender differences are examined with respect to start reaction times among elite swimmers at Swimming World Championships.

## Material and Methods

### Participants

The data consisted of 21 females and 23 males from the 2003 SWC, 22 females and 23 males from the 2005 SWC, 23 females and 24 males from the 2007 SWC, 22 females and 24 males from the 2009 SWC, 22 females and 23 males from the 2011 SWC, and 23 females and 23 males from the 2013 SWC. The different number of swimmers in each SWC can be explained by swimmers participating in two finals (as opposed to merely one) at the same SWC.

### Measures

The top six swimmers in each final 50 m event were targeted in the observational study. Each of those six swimmers participated in three consecutive events: the preliminary, semifinal, and final heats of their respective SWC. Each swimmer’s start reaction time was calculated as an average of the times of his or her three consecutive events. Athletes who took seventh and eighth place in the finals were excluded from the statistical analysis so as to minimize the cases that do not have maximum swimming start performance in the final part of the SWC. The effect of different motivation levels on the SRT in the preliminary heats, compared to the final was not considered in the present study.

### Procedures

Before proceeding, we present some necessary statistical terminology that was presented by Picquelle and Mier (2011). The primary sampling unit is “(…) an element within a sampling frame that is sampled and is statistically independent of other sampling units within the frame”. The sampling frame is “the collection of all elements (primary sampling units) accessible for sampling in the population of interest” (Picquelle and Mier, 2011). A single-unit design structure refers to observational studies in which the primary sampling unit is defined at only one scale (Hurlbert, 2013). The single-unit design structure was applied to the analysis of the protocols of the SWCs, wherein the swimmer was defined as a primary sampling unit and the swimming stroke final (male/female) event was defined as a sampling frame.

This study used a data set compiled from six SWCs during the years from 2003 to 2013. The source of the data was the Omega official timekeeper 2013 (http://www.omegatiming). Swimmers in the first six places in the final heats of four 50 m swimming strokes (viz., the freestyle, butterfly, breaststroke, and backstroke) were analyzed. The study was approved by the Ethical Committee of Ariel University (SP-15-2013, from August 5, 2013) with a waiver for the requirement of informed consent because the study involved the analysis of publicly available data.

#### Statistical Analyses

An unpaired two-tailed t-test was used when interval data sets from each SWC were evaluated. Before using the t-test, the Fisher-Snedecor F-test was used to confirm equal variability. The Satterthwaite’s approximate t-test was performed if variances were not confirmed to be equal. The descriptive statistics are presented as means (s) ± standard deviations (SD). The degrees of freedom (df), t-value-associated p-values, precise p-values, percent differences (Change%) between males and females and Cohen’s d-test were used to obtain the full statistical information. Change% was calculated using the ratio SRT_max_*100/SRT_min_-100. Neo-Fisherian significance assessments were used to interpret the signs and magnitudes of the statistical effects. The p-values were evaluated according to three-valued logic: “it seems to be positive” (i.e., there seems to be a gender difference), “it seems to be negative” (i.e., there does not seem to be a gender difference) and “judgment is suspended” regarding the gender difference (Hurlbert and Lombardi, 2009; 2012).

## Results

Gender differences among the top-sprint swimmers in six SWCs were statistically evaluated using a single-unit design structure (Table 2). Table 2 reflects the four separate groups: Freestyle (A), Butterfly (B), Breaststroke (C), and Backstroke (D). Table 2 also represents the two types of pooled groups: the pooling into a single group of all swimming strokes in one SWC (E) and the pooling into a single group of all swimming across the six SWCs (F).

For the freestyle, the GDs in SRTs seem to be positive for five SWCs. In contrast, it seems to be negative for one SWC (Table 2, A). As shown in Table 2 (B), for the butterfly, the GDs in SRTs seem to be positive for two SWCs, and they seem to be negative for two SWCs. In two SWCs, the judgments about gender differences are suspended. For the breaststroke, the gender differences in SRTs seem to be positive for two SWCs, whereas the gender differences in SRTs seem to be negative for four SWCs (Table 2, C). Table 2 (D) demonstrates that for the backstroke, five SWCs have gender differences in SRTs that seem to be negative, whereas in one SWC, the judgment relating to a gender difference in SRTs is suspended.

Table 2 (E) considers the effect on statistical differences regarding GDs that occurs as a consequence of ignoring naturally occurring groups through the pooling into a single group of all swimming strokes in one SWC. For two SWCs, the gender differences in start reaction times seem to be positive, whereas in one SWC, it seems to be negative. In three SWCs, the judgment about a gender difference in start reaction times is suspended.

Table 2 (F) shows that when we pool the four swimming strokes across the six SWCs, the gender difference in start reaction times seems to be positive.

## Discussion

The first goal of this study was to test the hypothesis that gender differences in start reaction times for elite sprint swimmers depend on the type of swimming stroke. Surprisingly, in the 50 m freestyle final events, gender differences in the start reaction times were found in five out of the six SWCs studied. In contrast, in the 50 m backstroke final events, gender differences in the start reaction times of five out of the six SWCs were not found. For both the 50 m butterfly and the breaststroke, gender differences in start reaction times yielded inconsistent statistical differences. At the present time, we lack both direct and indirect evidence regarding the different types of adaptations that may affect start reaction times among elite swimmers. However, in the scientific literature, unexpected gender differences have been noted. For example, at least three experimental studies have clearly shown females to have superior abilities to maintain balance compared with males (Kollegger et al., 1992; Ageberg et al., 2001; Park et al., 2013). Women also have previously been found to have better standing balance than men in the two-footed stance (Kollegger et al., 1992) and the single-limb stance (Ageberg et al., 2001). Recently, gender differences in elite short-track speed skaters have been studied; there, female athletes’ left and right static balance indexes were found to be significantly better than for male athletes (Park et al., 2013).

A second goal of this paper was to show that ignoring natural groupings can lead to erroneous conclusions. Four male/female swimming stroke final events within one SWC were pooled into a single group. We demonstrated that pooling the four swimming strokes in this manner resulted in erroneous conclusions, such as gender differences in start reaction times in the 2005 SWC and the 2009 SWC seeming to be positive, in the 2003 SWC seeming to be negative, and in the 2007 SWC, the 2011 SWC, and the 2013 SWC requiring inferential judgments to be suspended. In contrast, Pilianidis et al. (2012) pooled in one group three final (male/female) events for single Olympic Games: the 100 m and 200 m sprints and the 100/110 m hurdles. Strong evidence was found supporting the existence of gender differences in start reaction times (Pilianidis et al., 2012). However, the power of the statistical difference can be overestimated (Hurlbert, 2009; Picquelle and Mier, 2011). This overestimation occurs because athletes were pooled from different naturally created groups. This situation was identified as an example of sacrificial pseudoreplication (Hurlbert, 1984; 2013).

A third goal of this paper was to show that large sample sizes may lead to situations in which the power of the statistical difference is overestimated. For example, Tønnessen et al. (2013) statistically analyzed large samples of elite sprint athletes in the 100 m (males, *n* = 647; females, *n* = 540). The large sample size was obtained by pooling all members of each gender from four AWCs and four AWYCs. We calculated that the gender difference in start reaction times was approximately 6%. It was commonly suggested that despite an occurrence of a small absolute difference in start reaction times, the chosen p-value (p <0.01) offered strong evidence of a gender difference. To explain pooling the data set across members of the same gender, Tønnessen et al. (2013) noted that “(…) the data presented in this study were collected under highly controlled and standardized procedures, and we believe they are reliable”. In the present study, we pooled within each gender all swimming strokes and all six SWCs from 2003 to 2013. As a result, the degrees of freedom increased from 10 to 286. A very low *p*-value (*p* = 0.00004) offered strong evidence for the existence of a gender difference in the start reaction times for the elite sprint swimmers. However, we suggest that this finding is better attributed to the large sample size rather than to differences in the neuromuscular physiology of the swimmers.

Recently, ignoring the natural groupings of objects has been shown to result in artificially inflated degrees of freedom. Such inflation can lead to the illusion of a more powerful statistical difference. This error in the statistical analysis exemplifies sacrificial pseudoreplication (Picquelle and Mier, 2011; Hurlbert and Lombardi, 2009; Hurlbert, 2013). In this context, sacrificial pseudoreplication occurs upon pooling into one group the performances of elite sprint swimmers’ final events at a single SWC from four swimming stroke groups - viz., freestyle, butterfly, breaststroke, and backstroke - or upon pooling all swimmers from the four swimming stroke final events across all SWCs.

In summary, the following three statistical assumptions were presented: (i) swimmers of 50 m final events at SWCs can be naturally divided into four (freestyle, butterfly, breaststroke, and backstroke) swimming strokes, (ii) pooling all swimmers of the same gender leads to sacrificial pseudoreplication, and (iii) the large sample size contributes to overestimation of statistical differences. It was shown that using a single-unit design structure - i.e., defining a swimmer at only one scale - can remedy pseudoreplication. Based on these three assumptions, gender differences were found in the start reaction times for the freestyle sprint swimmers. In contrast, no gender differences were found in the start reaction times for the backstroke sprint swimmers. Inconsistent gender differences in the start reaction times were found for both butterfly and backstroke sprint swimmers. The contrasting start reaction time gender difference observed between the freestyle and backstroke final events of the SWCs may be associated with different types of gender-related adaptations to swimming performances.

## Figures and Tables

**Table 1:** Example of Non-demonic intrusions in Swimming World Championships

Year	Place	Starting Block Type
The 10th FINA World Championships 2003	Barcelona, Spain	Regular
The 11th FINA World Championships 2005	Montreal, Canada	Regular
The 12th FINA World Championships 2007	Melbourne, Australia	Track Start with handrails
The 13th FINA World Championships 2009	Rome, Italy	Regular
The 14th FINA World Championships 2011	Shanghai, China	Track Start
The 15th FINA World Championships 2013	Barcelona, Spain	Track Start

**Table 2:** Average gender differences in start reaction times, Mean ± standard deviation (SD).

Stroke	SWC	Males	Females	df	t test	Change %	d test	p
Freestyle (A)	2003	0.77±0.05	0.76±0.02	10	0.492	1.31	0.31	0.633^b^
	2005	0.68±0.03	0.79±0.04	10	−5.285	16.18	3.34	0.0004^a^
	2007	0.71±0.03	0.77±0.04	10	−2.857	8.45	1.81	0.017^a^
	2009	0.71±0.02	0.76±0.02	10	−3.766	7.04	2.38	0.004^a^
	2011	0.66±0.01	0.70±0.03	10	−2.977	6.06	1.88	0.014^a^
	2013	0.65±0.04	0.71±0.04	10	−2.798	9.58	1.77	0.019^a^

Butterfly (B)	2003	0.73±0.02	0.78±0.03	10	−3.503	6.85	2.22	0.006^a^
	2005	0.73±0.06	0.76±0.07	10	−0.736	4.11	0.47	0.479^b^
	2007	0.71±0.05	0.78±0.08	10	−1.686	9.86	1.07	0.123^c^
	2009	0.72±0.02	0.76±0.03	10	−3.058	5.56	1.93	0.012^a^
	2011	0.67±0.02	0.69±0.04	10	−1.034	2.99	0.65	0.325^b^
	2013	0.65±0.02	0.69±0.04	10	−2.045	5.36	1.29	0.068^c^

Breaststroke (C)	2003	0.77±0.04	0.76±0.04	10	0.144	1.31	0.09	0.889^b^
	2005	0.71±0.04	0.75±0.05	10	−1.245	5.63	0.78	0.241^b^
	2007	0.71±0.07	0.76±0.05	10	−1.346	7.04	0.85	0.207^b^
	2009	0.71±0.03	0.77±0.03	10	−3.257	8.45	2.06	0.009^a^
	2011	0.67±0.03	0.74±0.04	10	−3.257	10.45	2.06	0.009^a^
	2013	0.68±0.03	0.69±0.06	10	−0.386	1.81	0.24	0.708^b^

Backstroke (D)	2003	0.67±0.05	0.64±0.02	10	1.678	4.69	1.06	0.124^c^
	2005	0.68±0.05	0.72±0.05	10	−1.326	5.88	0.84	0.215^b^
	2007	0.71±0.04	0.67±0.05	10	1.245	5.97	0.79	0.242^b^
	2009	0.61±0.08	0.66±0.08	10	−1.121	8.20	0.71	0.289^b^
	2011	0.62±0.07	0.63±0.05	10	−0.010	1.61	0.01	0.992^b^
	2013	0.61±0.07	0.61±0.03	10	0.150	1.19	0.09	0.883^b^

Pooling year (E)	2003	0.74±0.06	0.74±0.06	46	0.027	0.00	0.01	0.979^b^
	2005	0.70±0.05	0.75±0.06	46	−3.389	7.68	1.00	0.002^a^
	2007	0.71±0.05	0.75±0.07	46	−1.952	5.63	0.58	0.057^c^
	2009	0.69±0.06	0.74±0.06	46	−2.589	7.42	0.76	0.013^a^
	2011	0.66±0.04	0.69±0.05	46	−2.010	4.55	0.31	0.050^c^
	2013	0.64±0.05	0.67±0.06	46	−1.629	3.95	1.03	0.110^c^

Pooling years (F)		0.69±0.06	0.72±0.07	286	4.161	1.08	0.49	0.00004^a^

Percent (%) change was calculated as the ratio SRT_max_^*^100/SRT_min_-100. The p-values were evaluated according to three-valued logic:

a - it seems to be positive,

b - it seems to be negative, and

c -judgment is suspended.
